# Computed tomography-guided radiofrequency ablation for palliation of a painful supraclavicular soft-tissue metastasis invading the brachial plexus

**DOI:** 10.3892/ol.2013.1577

**Published:** 2013-09-12

**Authors:** ARISTOTELIS KECHAGIAS, SPIROS DELIS, CHRISTOS DERVENIS, PETROS MANIATIS, JOHN PAPAILIOU

**Affiliations:** 1 First Department of Surgery, Konstantopouleion-Agia Olga General Hospital, Athens 142-33, Greece; 2 Department of Computed Tomography and Invasive Radiology, Konstantopouleion-Agia Olga General Hospital, Athens 142-33, Greece

**Keywords:** soft tissue metastasis, supraclavicular, radiofrequency ablation, palliation, computed tomography-guided

## Abstract

The present study describes a case of a painful supraclavicular soft-tissue metastasis of a skin melanoma invading the brachial plexus in a 38-year-old male. The patient was treated twice with radiofrequency ablation (RFA) under computed tomography (CT) guidance, which caused tumoral necrosis. The patient was originally referred with a 7-cm metastasis in the right supraclavicular fossa, which caused intractable pain and a degree of numbness. These symptoms were unresponsive to chemotherapy and radiotherapy and the pain was not controlled using narcotic analgesics. The lesion was treated with CT-guided RFA causing necrosis, relieving the pain and sparing the patient from using analgesics. The pain recurred 19 months thereafter and a CT scan revealed an 8-cm mass in the right supraclavicular space. The patient underwent repeat CT-guided RFA, which reduced the pain to a level that was controlled with minor oral analgesics. In conclusion, in this case of a painful supraclavicular soft-tissue metastasis invading the brachial plexus, which was intractable to chemotherapy and radiotherapy, RFA was feasible and offered substantial palliation of the symptoms, freedom from the use of narcotic analgesics and improvements to the quality of life.

## Introduction

Computed tomography (CT)-guided radiofrequency ablation (RFA) is a widespread technique that is used to treat selected liver malignancies where surgery is contraindicated or as an adjunct to it. During recent years, the indications for RFA application have been enhanced to include the palliative treatment of cancer-related pain, particularly in patients with bone malignancies (1). However, to date, few studies have dealt with the use of RFA to relieve pain from soft-tissue malignancies that are refractory to conventional therapeutic modalities (2–5). The present study aimed to describe the outcome following the use of RFA for the palliative management of a painful supraclavicular soft-tissue metastasis of a skin melanoma invading the brachial plexus. Written informed consent was obtained from the patient's family.

## Case report

A 38-year-old Caucasian male was referred to the Department of Computed Tomography and Invasive Radiology, Konstantopouleion-Agia Olga General Hospital (Athens, Greece) due to a painful right supraclavicular soft-tissue metastasis of a skin melanoma that was unresponsive to conventional therapy. The patient had undergone a surgical excision of the skin melanoma, which was located at the right scapular area, and a simultaneous resection of the ipsilateral axillary metastatic nodes was performed 17 months earlier, followed by a 6-month regimen of immunotherapy with interferon-α and chemotherapy. At 14 months post-surgery, pain and a degree of numbness gradually developed in the right upper limb. Magnetic resonance imaging was performed and revealed a metastatic mass of 7-cm in diameter in the right supraclavicular area. The patient was subsequently administered 10 sessions of external beam radiation therapy for a total dose of 48 Gy that proved ineffective to relieve the symptoms or reduce the size of the mass. Furthermore, the pain was unresponsive to aggressive therapy with escalating doses of narcotic analgesics. A post-radiotherapy CT-scan confirmed a 7-cm mass that occupied the right supraclavicular space. Following a surgical consultation, the lesion was considered to be inoperable due to brachial nerve plexus infiltration, as indicated by the symptomatology.

**Figure 1:**
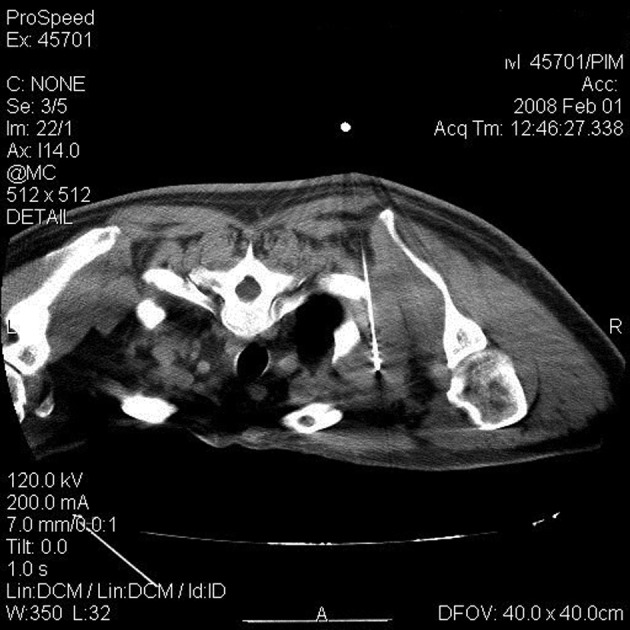
Computed tomography (CT)-guided radiofrequency ablation (RFA) probe in the right supraclavicular soft-tissue mass (diameter, 6×7 cm). The scan was taken with the patient in the prone position.

**Figure 2:**
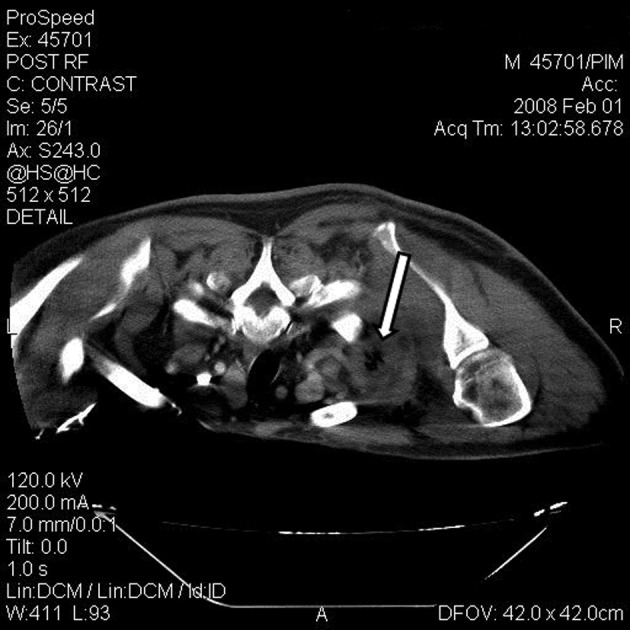
Visualization of coagulation necrosis in the centre of the mass (arrow) with a contrast-enhanced computed tomography (CT) scan immediately after the radiofrequency ablation (RFA) with the patient in prone position.

Percutaneous RFA of the tumor under CT-guidance was therefore decided upon. The procedure was performed under local anesthesia assisted by oral benzodiazepines. Slices (5 mm) were obtained using spiral CT. An Electrotom HiTT^®^ 106 (Berchtold Holding GmbH, Tuttlingen, Germany) and a high-frequency-induced thermotherapy needle applicator (EZ 703-20; diameter, 2.0 mm; shaft length, 150 mm; and electrode length, 20 mm) perfusable with normal saline solution were used. The patient was placed in a prone position and the needle tip was proceeded gradually from the posterior side of the trapezius muscle to the tumor mass. Care was taken to gently change the direction of the shaft in case of stimulation of the brachial plexus by contact. When the needle tip was positioned at the center of the lesion (Fig. 1), 50 W radiofrequency energy was applied for a total duration of 10 min with an intermittent interval time of 9 min at 5 min. A CT image was obtained at this interval to control tumor necrosis. Subsequent dual-phase CT axial images at the end of the procedure confirmed thermal necrosis of the tumor (Fig. 2). The patient remained hospitalized for 24 h.

**Figure 3:**
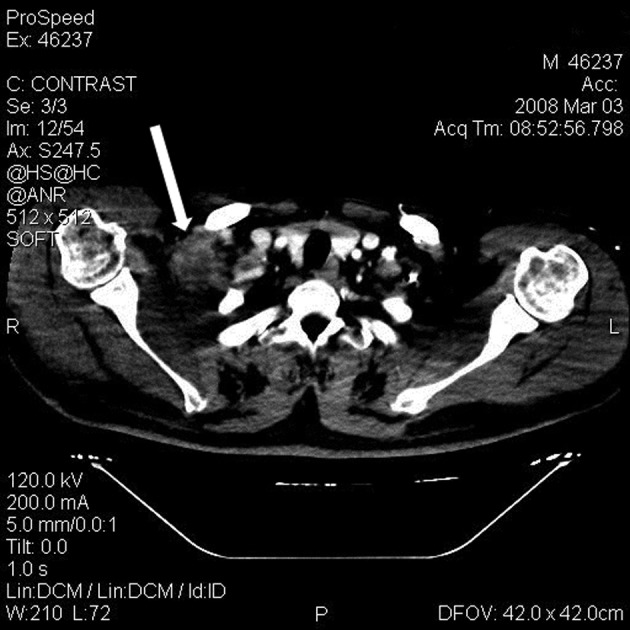
Post-ablation shrinkage of the mass (arrow) by half its size (3 cm) at the one-month follow-up computed tomography (CT) scan with the patient in supine position.

Four days later, the pain decreased to a level that was lower than prior to the RFA and the patient discontinued the narcotic medication. The degree of upper limb numbness remained unaffected. Overall, the patient demonstrated an improvement in the daily quality of life following the thermal ablation of the lesion. The overall satisfaction for pain control following the procedure was 8/10 according to the visual analogue scale (VAS). Accordingly, a CT scan at the one-month post-RFA follow-up revealed shrinkage of the initial mass to 3 cm in diameter (Fig. 3). The patient declined to undergo a control CT scan at 6 months due to a lack of significant pain.

**Figure 4:**
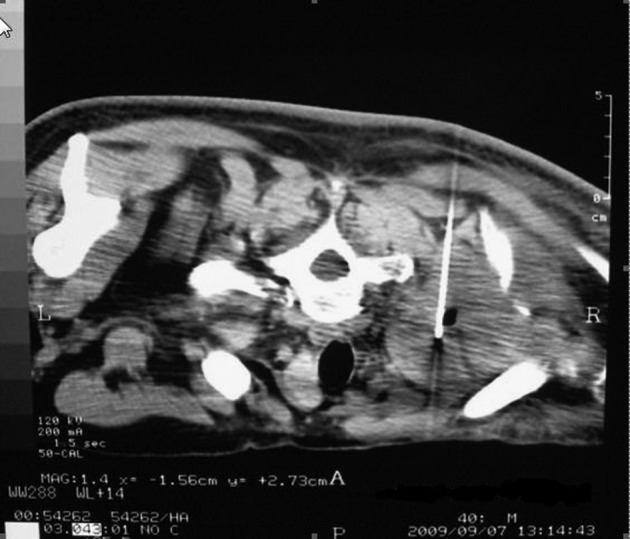
Repeat radiofrequency ablation (RFA) in the right supraclavicular soft-tissue mass (diameter, 7×8 cm) with the patient in prone position.

**Figure 5:**
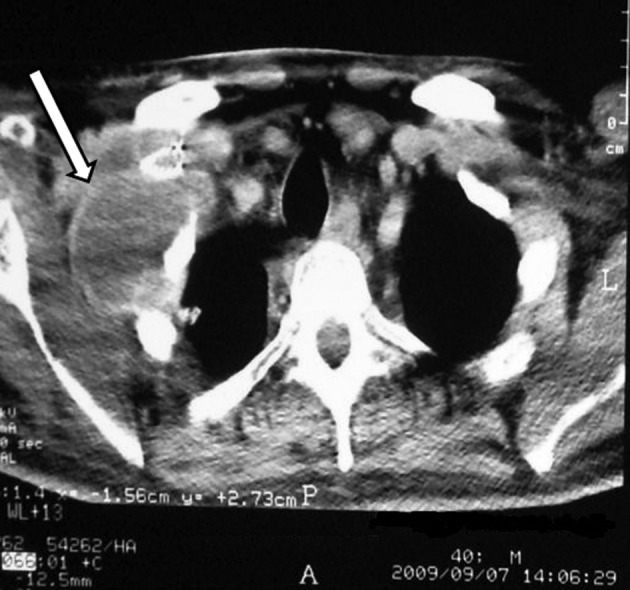
Visualization of the cystic transformation of the mass (arrow) with a contrast-enhanced computed tomography (CT) scan immediately after the repeat radiofrequency ablation (RFA) with the patient in supine position.

At 19 months post-RFA, the patient presented with a gradual reappearance of the pain in the same area. However, the limited upper limb motility remained unchanged. A CT scan was obtained that revealed an 8-cm soft-tissue local recurrence with identical imaging signs as prior to the RFA. A repeat RFA was performed in the same manner as described previously (Figs. 4 and 5) and a follow-up CT scan at one month post-RFA revealed mass necrosis without shrinkage. Although the pain relief following the second RFA was less than following the initial procedure, the patient confirmed a clinical benefit that corresponded to 6/10 satisfaction for pain control according to the VAS. At 2 months after the repeat RFA, the patient presented with tolerable pain that was easily controlled with minor oral analgesics.

## Discussion

Cancer-related pain and narcotic analgesic dependency is of major concern in the quality of life of patients with soft-tissue metastasis that involves a major nerve plexus. Several treatment modalities, including chemotherapy, radiotherapy and when possible, surgical excision, are employed to control the disease and its symptoms (6). However once they become ineffective, escalating doses of narcotics are required (7). The present study describes a patient with a supraclavicular soft-tissue metastasis invading the brachial plexus, which caused severe debilitating symptoms of pain and numbness in the upper limb. The lesion was unresponsive to chemotherapy and radiotherapy, and an effective surgical excision was not considered feasible due to nerve infiltration. As a result, the patient required considerable doses of strong opioids that further compromised his quality of life.

According to the present case and the existing literature (2–5), RFA appears efficient in the palliation of symptoms of painful soft-tissue metastatic malignancies when other therapies are ineffective. Sanou *et al*(5) reported a series of 12 patients with painful primary or secondary soft-tissue neoplasms in various locations of the body who were treated with RFA and achieved partial or complete response in the short- and the long-term. In particular, a patient with a 10-cm metastatic bronchial cancer in the scapular region involving the brachial plexus had 60% short-term palliation of the pain according to the VAS. The durability of the effect of the RFA was not documented in this case since the patient succumbed due to complications of the disease. RFA is a minimally invasive, repeatable and low-cost procedure. In numerous cases, RFA may be performed under local anesthesia and has the advantage of a short hospital stay. RFA ablation relieved the present patient from narcotic analgesic use and side-effects, resulting in a substantial improvement in the quality of life. The palliative effect of RFA is not permanent, but may provide relief for a significant period of time, as symptoms in this case only recurred after 19 months.

However, the value of RFA to treat painful metastasis has not been studied sufficiently in order to assess the exact role of the procedure in palliation and to standardize its precise therapeutic indications. To the best of our knowledge, this is the first case that is described in the literature with regard to the application of RFA in the supraclavicular fossa to alleviate neoplastic compressive symptoms. Further research is required to evaluate the effectiveness of RFA in relieving pain from soft-tissue metastasis in this area as a first-line approach compared with chemoradiation and/or medical treatment with narcotic analgesics. RFA is therefore a promising method, but one that currently should be used for selected cases where conventional therapies have failed. Furthermore, the present study demonstrated that the procedure may be safely applied to a disease that is localized to the supraclavicular space. Likewise, RFA may be beneficial in the palliation of metastases from other primary cancers that commonly arise there.

In conclusion, in the present case of painful supraclavicular soft-tissue metastasis invading the brachial plexus, RFA proved to be feasible and offered substantial palliation of the symptoms, freedom from narcotic analgesic use and improvements to the quality of life. Further investigation is essential for an improved definition of the role of RFA in the palliation of metastatic disease of the supraclavicular fossa.
